# Plasmonic Properties of Icosahedral-Seeded Gold Nanostars

**DOI:** 10.1021/acs.jpcc.5c07756

**Published:** 2026-01-20

**Authors:** Oliver Leal de Castro Prioli, Debora Ferrari, Laura Fabris, Daniel Ugarte, Diego Pereira dos Santos

**Affiliations:** † Institute of Chemistry, State University of Campinas, Campinas 13083-862, Brazil; ‡ Department of Applied Science and Technology, Politecnico di Torino, Turin 10129, Italy; ¶ Institute of Physics Gleb Wataghin, State University of Campinas, Campinas 13083-859, Brazil

## Abstract

Gold nanostars (AuNSs) exhibit rich plasmonic responses that are highly sensitive to the number, orientation, and relative length of their legs. Here, we use the boundary element method to investigate the optical properties of experimentally realistic AuNSs grown from icosahedral seeds. The simulated extinction spectra reveal three main plasmonic resonances: a high-energy, radial-like mode with charge oscillations from the core to the leg tips (mode #1, 700–900 nm), an intermediate mode (mode #2, ∼1000–1200 nm) that emerges when a shorter leg is present, and a low-energy dipolar mode (mode #3, ∼1300 nm) dominated by coupling between oppositely oriented legs relative to the core. Charge-density maps show that modes #1 and #2 arise from hybridization between the single-short-leg plasmon and the collective resonance of the legs originated from the same plane of the icosahedral core. Electron energy-loss spectroscopy simulations confirmed the radial-like nature of this collective mode. Near-field calculations show tip-localized SERS enhancement factors up to ∼10^8^, with modes #2 and #3 producing the strongest hot spots. These results establish structure–property relationships for applications such as ultrasensitive detection of a given analyte.

## Graphical abstract

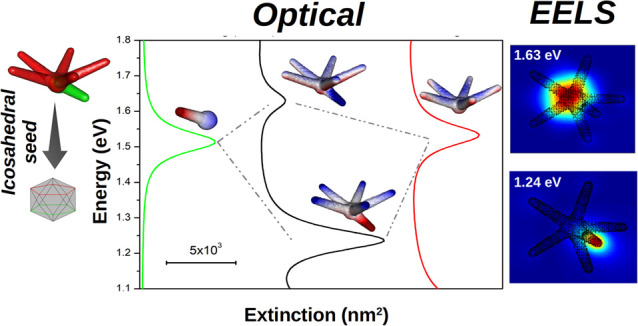

## Introduction

Metallic nanoparticles exhibit optical properties that differ significantly from their bulk counterparts due to the excitation of localized surface plasmon (LSP) modes at specific resonance energies. These resonances are strongly dependent on nanoparticle morphology and the surrounding chemical environment.^1,2^ Among such nanostructures, gold nanostars (AuNSs) have attracted considerable attention^3–7^ as highly asymmetric plasmonic nanoparticles whose optical response can differ substantially from that of more symmetric counterparts, such as nanospheres, nanorods, or nanobipyramids.

While the plasmonic resonances of symmetric nanoparticles are often dominated by a bright dipolar mode accompanied by a limited number of low-order multipolar modes, highly asymmetric structures with multiple branches support a much richer and more complex plasmonic response, which may involve the coupling and hybridization of multiple plasmonic modes associated with individual branches (or spikes, legs). In AuNSs, the plasmonic response can be effectively tuned by controlling the number, length, and spatial arrangement of the legs.^8^ Furthermore, the sharp curvatures at the leg tips give rise to intense local electromagnetic field enhancements, which are central to applications in enhanced spectroscopies, such as surface-enhanced Raman scattering (SERS).^7,9–12^

The precise morphological control of AuNSs, however, remains a challenging task. Atta et al.^8^ investigated the synthesis of six-branched AuNSs by varying the concentrations of surfactant (Triton X), reducing agent (ascorbic acid), silver nitrate (AgNO_3_) and gold seeds. In particular, they demonstrated that AgNO_3_ is essential for nanostar formation, with polyhedral nanoparticles obtained in its absence. Variations in these synthesis parameters were found to produce profound changes in the extinction spectra in the 400–1100 nm range. A remarkable observation was a time-dependent evolution of the plasmonic response: an initial redshift of the main band up to ∼1000 nm, followed by a blueshift for longer reaction times (above 5 min). The redshift was attributed to leg elongation, whereas the blueshift was explained by surface diffusion processes reshaping the legs.

Building upon the study of six-branched AuNSs, Corrêa et al. recently performed a four-dimensional scanning transmission electron microscopy (4D-STEM) characterization of an individual AuNS.^13^ 4D-STEM is a powerful technique that provides detailed structural and crystallographic information at the nanoscale.^14,15^ The analysis revealed an icosahedral core, with the legs identified as high-aspect-ratio decahedral rods attached at the icosahedral vertices (5-fold axes). Based on these observations, a growth mechanism was proposed in which surfactant layer “unzipping” leads to legs growth on adjacent icosahedral vertices.

Recently, Corrêa et al. performed boundary element method (BEM) simulations to model the optical response of different AuNSs with structures dictated by an icosahedral seed core.^16^ The results showed very good agreement with the experimental extinction spectra, highlighting the role of out-of-plane legs in the observed features. This was demonstrated by comparing a model composed of five legs oriented in space along icosahedral 5-fold axes with a planar structure containing the same number of legs, which could correspond to a decahedral seed. The icosahedral-based nanostar exhibited a strong plasmonic peak around 800 nm that was absent in the planar configuration. In planar structures, two low-intensity peaks appeared in the 600–700 nm range, which were also present in the icosahedral-based structure, but with higher intensities.

Tsoulos and Fabris^11^ performed finite element method (FEM) simulations of Au nanostars with varying shapes and numbers of legs, and interpreted the higher-energy modes (600–700 nm) as coupled bulk and surface propagating plasmons. The authors showed that the intensities of these modes can be attenuated in planar leg arrangements, especially in colinear configurations, due to interference effects.

The emergence of a new plasmonic mode in the icosahedral-based nanostar is a particularly intriguing result, as it can be exploited to establish morphology–property correlations within a given experimental setup. However, for such nanostar geometry, no clear explanation of these observations has been provided, nor has a detailed analysis of the optical response been performed, particularly concerning the low-energy modes in the near-infrared region, which are highly relevant for biological applications, especially in the 800–1400 nm range, where tissue constituents exhibit low absorption, allowing deeper radiation penetration.^17^ In the present work, we conduct a comprehensive investigation of the plasmonic properties of gold nanostars—including mode assignments under optical and electron excitations, as well as near-field distributions, focusing on structures consistent with the experimental observations from 4D-STEM characterization.^16^

## Methods

The plasmonic properties of gold nanostars (AuNSs) were simulated using the boundary element method (BEM),^18^ as implemented in the MNPBEM17 toolbox developed by Hohenester and Trügler.^19^ Figure 1 shows representative BEM models (first row) of icosahedral-based AuNSs that were experimentally characterized in a recent investigation.^16^

**Figure 1:**
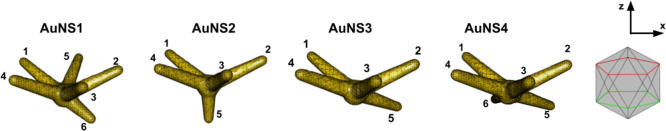
BEM models used in the simulations of icosahedral-seeded AuNSs, considering a spherical Au core with a diameter of 25 nm and cone-like legs of 65 nm in length, with base and tip diameters of 15 and 10 nm, respectively. The tips were modeled by hemispherical caps with a diameter of 10 nm. Each structure (AuNS1-AuNS4) represents an experimentally characterized AuNS from a recent investigation.^16^ The icosahedral geometry associated with the core of the experimental particles is also shown, together with the Cartesian axes notation used to describe the AuNS orientations. The leg numbering aims at facilitating the morphological differences between the AuNSs in this set.

The synthesis of AuNSs typically yields a variety of morphologies differing in the number and length of their legs. The structures presented in Figure 1 were identified by Corrêa et al.^16^ through 4D-STEM characterization of the synthesized samples, following the methodology previously reported.^13^ The four nanostar morphologies were interpreted as resulting from anisotropic leg growth along the radial directions defined by the vertices of an icosahedral Au seed (see schematic representation in Figure 1). To reproduce the experimental morphologies,^16^ the central core was modeled as a 25 nm Au sphere, while the legs were approximated as truncated cones with base and tip diameters of 15 and 10 nm, respectively. The orientation of each BEM model relative to the Cartesian *x*- and *z*-axes is also indicated in Figure 1, which additionally shows the nanoparticles after rotations to facilitate comparison with the TEM images reported in the experimental investigation.^16^ For clarity, the main structural parameters obtained from the 4D-STEM analysis are summarized in Table 1.

**Table 1:** Summary of Structural Parameters for the Modeled AuNSs in Figure 1 ^a^

nanostar	number of legs	leg length (nm)	leg diameters (nm)
AuNS1	6	5 legs: 65; 1 leg: 40	15 (base)/10 (tip)
AuNS2	5	4 legs: 65; 1 leg: 40	15/10
AuNS3	5	all 65	15/10
AuNS4	6	all 65	15/10

a The core diameter was fixed at 25 nm in all cases.

The AuNS geometries used in the BEM calculations were generated by describing the particle surface as a point cloud, which was converted into triangular surface meshes using MeshLab^20^ and routines implemented in the R programming language.^21^ In this process, slight deviations from the nominal particle dimensions occurred due to the mesh construction. For instance, the simulated core radius was approximately 24 nm, while the two main leg lengths were approximately 66 and 42 nm, instead of the nominal 65 and 40 nm. These small discrepancies do not affect the qualitative discussion and are therefore reported as the effective dimensions throughout the manuscript. Whenever size-dependent effects are analyzed, the actual leg dimensions are considered.

Within MNPBEM17, absorption, scattering, and extinction cross sections were calculated (see Supporting Information, SI). Both gold and the surrounding water medium were modeled with μ = 1. For water, a constant dielectric function of 1.777 was used,^22^ while for gold, the experimental dielectric data of Johnson and Christy^23^ were employed. This data set is widely used for plasmonic simulations and better agreement with experimental results^24^ can be achieved when alternative data sets such as those of Palik^25^ or McPeak et al.^26^ are used, particularly for wavelengths above 700 nm. The surface mesh was refined at the leg ends to accurately capture local field enhancements. The first nanostar model (AuNS1) was discretized into approximately 7800 surface elements. To reduce computational cost, the hierarchical matrix solver implemented in MNPBEM17 ^27^ was employed for efficient evaluation of the integral equations S3 and S4 presented in the SI.

The polarization-averaged optical response of the AuNSs was initially evaluated by performing simulations for 12 excitation configurations,^28^ each defined by a specific wavevector–polarization pair (*k*_*i*_, *E*_*j*_):

$$\left(k,E\right)=\{\begin{array}{l} \left(k_{x},E_{y}\right)\\ \left(k_{-x},E_{y}\right)\\ \left(k_{x},E_{z}\right)\\ \vdots \\ \left(k_{-z},E_{y}\right) \end{array}$$ 1

where *k*_*i*_ and *E*_*j*_ denote the propagation and polarization directions along the Cartesian axes. For AuNS1, different propagation directions with the same polarization (e.g., (*k*_*x*_, *E*_*y*_), (*k*_–*x*_, *E*_*y*_), (*k*_*z*_, *E*_*y*_), (*k*_–*z*_, *E*_*y*_)) yielded negligible differences (see SI, Figure S1). Therefore, to reduce the computational cost for AuNS2, AuNS3 and AuNS4, the simulations were restricted to three representative configurations:

$$\left(k,E\right)=\{\begin{array}{l} \left(k_{-z},E_{x}\right)\\ \left(k_{-z},E_{y}\right)\\ \left(k_{-x},E_{z}\right) \end{array}$$ 2

Electron energy-loss spectroscopy (EELS) simulations were also carried out using the MNPBEM17 toolbox, considering an electron beam with an energy of 200 keV and a beam width of 0.2 nm as the excitation source. The high kinetic energy ensures that the associated energy losses remain small perturbations to the electron trajectory.^29^ All EELS simulations were performed in a homogeneous dielectric environment (water) for direct comparison to the optical excitation.

## Results and Discussion

### Optical Properties of Au Nanostar Structures

All AuNSs share the presence of legs #1–#4 (numbering in Figure 1), which extend along the 5-fold symmetry axes from the apexes of the upper plane, highlighted in red in the icosahedral seed scheme of Figure 1. Hereafter, we refer to these as “upper-plane legs”, which does not imply that they lie on the same physical plane, but rather that they originate from vertices belonging to the same plane of the icosahedral seed. Leg #2 lies in the *xz*-plane indicated in Figure 1. Among the simulated nanostars, AuNS2, AuNS3 and AuNS4 exhibit only four upper-plane legs, whereas AuNS1 displays five, with an additional shorter leg (#6, ∼40 nm) extending from the lower plane (highlighted in green in Figure 1). AuNS4 also presents six legs (all 65 nm in length), with two legs originating from vertices of the lower plane. AuNS2 and AuNS3 each display five legs but with distinct configurations: in AuNS3, the fifth leg arises from the lower plane, while in AuNS2 the shorter leg (∼40 nm) is oriented along the *z*-axis.

Figure 2 shows the simulated extinction spectra for all AuNS structures under different incident polarizations (Figure 2A) and their polarization-averaged spectra (Figure 2B). The inset highlights the higher-energy region of the spectra also reported by Corrêa et al.^16^

**Figure 2:**
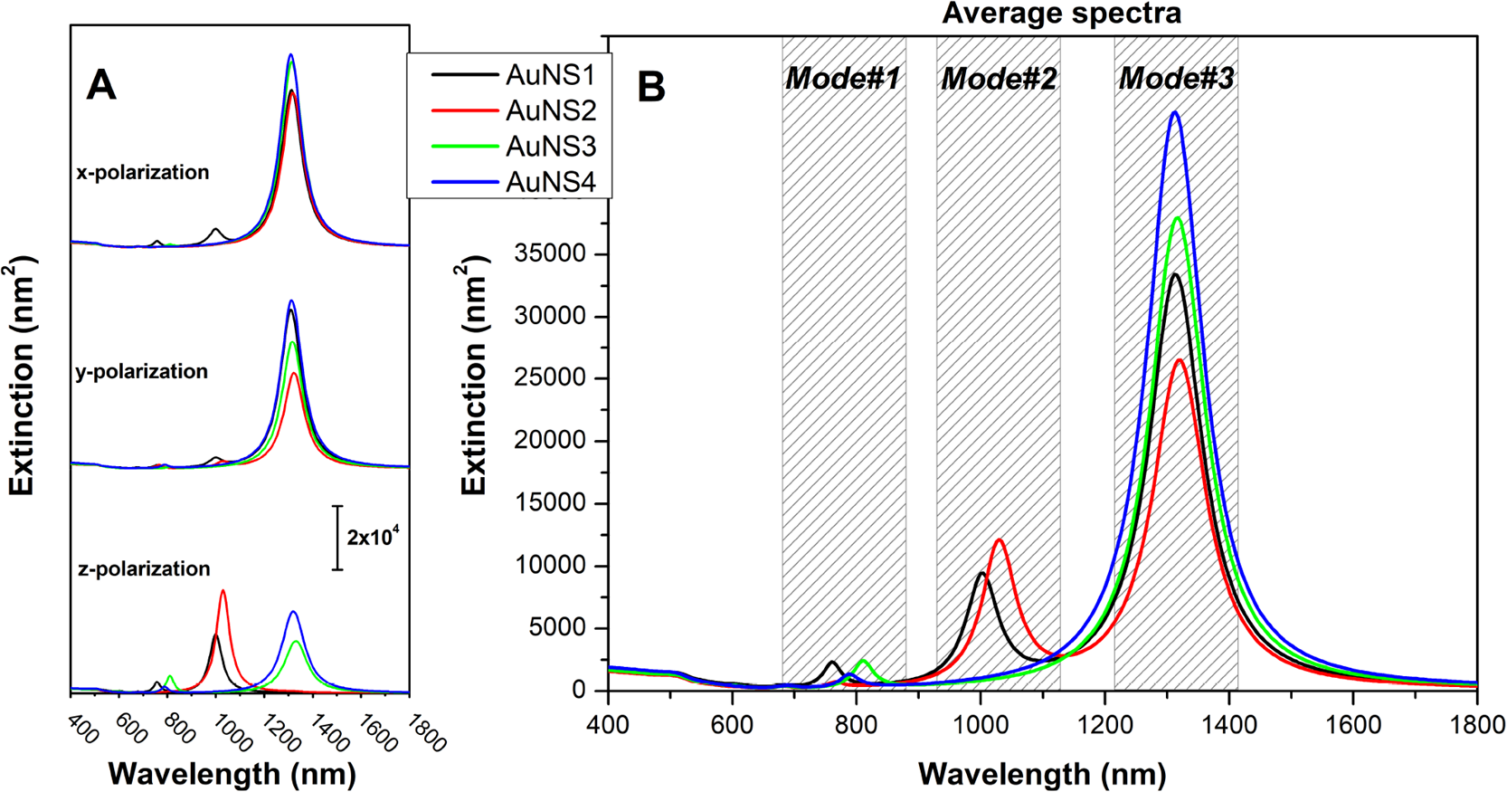
BEM-simulated extinction spectra for the four AuNS structures shown in Figure 1. (A) Spectra under different incident electric-field polarization directions, following the axes and AuNS orientations defined in Figure 1. (B) Polarization-averaged spectra for each structure. Three plasmonic modes (modes #1–#3) are highlighted by hatched boxes.

All AuNSs exhibit two well-defined plasmon resonance modes: one in the 700–900 nm range (mode #1) and another at approximately 1300 nm (mode #3). Additional low-intensity peaks are observed near 600 and 680 nm, showing minimal variation among the different AuNSs. These weaker features are consistent with the results of Tsoulos and Fabris,^11^ who assigned them to harmonics of a fundamental standing-wave mode associated with the coupling between bulk and surface-propagating plasmons. As discussed in their work, reduced intensities of these harmonics arise from interference effects that become more pronounced as the number of legs increases. Given their minor contribution to the overall extinction, the discussion below focuses primarily on the dominant resonance in this region (mode #1), which was also identified in our recent investigation.^16^

The resonance wavelength of mode #3 remains nearly unchanged across the different AuNS structures, with variations mainly in relative intensity. In contrast, mode #1 exhibits pronounced changes in both spectral position and extinction cross-section, as highlighted in the inset of Figure 2B. For instance, comparison between AuNS3 and AuNS4—structurally similar except for the additional 65 nm leg in the lower plane of AuNS4—reveals a blue shift of the resonance accompanied by a decrease in extinction cross-section. A similar trend is observed when comparing AuNS1 and AuNS2: the presence of an additional leg in AuNS1 also shifts the resonance to higher energies, though in this case the extinction cross-section increases. These results indicate that mode #1 is highly sensitive to structural variations, exhibiting a general blue shift as the number of legs increases. These blue shifts may also contribute, together with the reshaping effects suggest by Atta et al.,^8^ to the experimentally observed spectral evolution during the synthesis. Overall, these findings demonstrate that the optical response of icosahedral-seeded AuNSs is strongly governed by the interplay between leg number and length, with mode #1 acting as a particularly sensitive probe of structural variations.

Beyond these main features, AuNS1 and AuNS2 exhibit an additional resonance around 1000 nm (mode #2), which is absent in AuNS3 and AuNS4. This mode is most clearly observed under *z*-axis polarization of the incident field, which also produces the strongest response for mode #1. Interestingly, for this polarization, mode #3 is suppressed in AuNS1 and AuNS2, whereas it dominates the extinction response under *x*-axis polarization.

Figure 3 shows the surface charge density distributions (see SI file and the discussion of eqs S6 and S7) for the four AuNS structures at the three main plasmon resonances (modes #1–#3). Only the polarizations leading to the strongest extinction response for each mode are displayed.

**Figure 3:**
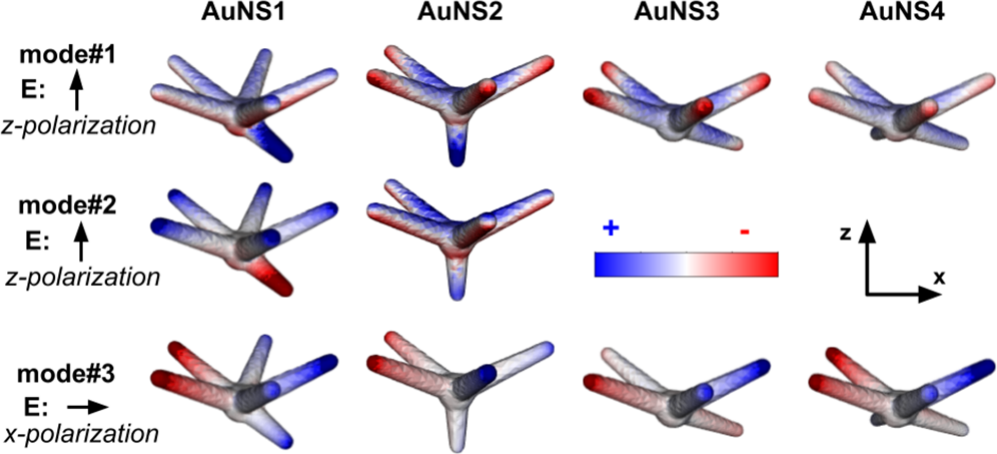
Surface charge density distributions for the four AuNS structures. Columns correspond to the different nanostars, and rows to modes #1–#3. Results are shown for *z*-axis polarization (modes #1 and #2) and *x*-axis polarization (mode #3).

The surface charge density distributions for mode #3 reveal a dipolar-like coupling between legs positioned on opposite sides of the core along the *x*-axis, with the strongest contributions arising from upper-plane legs. The large extinction cross-section observed under *x*-polarization can be attributed to the presence of leg #2 in the *xz*-plane, which reinforces the overall dipole moment of the plasmon mode and thereby enhances its coupling with the radiation field.

Modes #1 and #2 display markedly different charge distributions compared to mode #3. Opposing charge distributions on legs grown from the same plane are no longer observed. Instead, mode #1 primarily arises from the coupling between the core and each leg, producing similar charge densities across most legs. Exceptions include the shorter 40 nm legs (#6 in AuNS1 and #5 in AuNS2), which alter the charge distribution pattern. Comparison of modes #1 and #2 in AuNS1 also indicates a polarization change involving the shorter leg, suggesting that modes #1 and #2 may originate from hybridization between this shorter leg and the plasmonic response of the remaining upper-plane legs.

To further investigate these observations, we modeled AuNSs with varying numbers of upper-plane legs. The polarization-averaged extinction spectra and the corresponding charge distributions are shown in Figure 4. All structures share the same core size (25 nm) and leg length (65 nm), with an additional simulation of a single-leg structure of 40 nm in length, which was included for comparisons (dashed black line).

**Figure 4:**
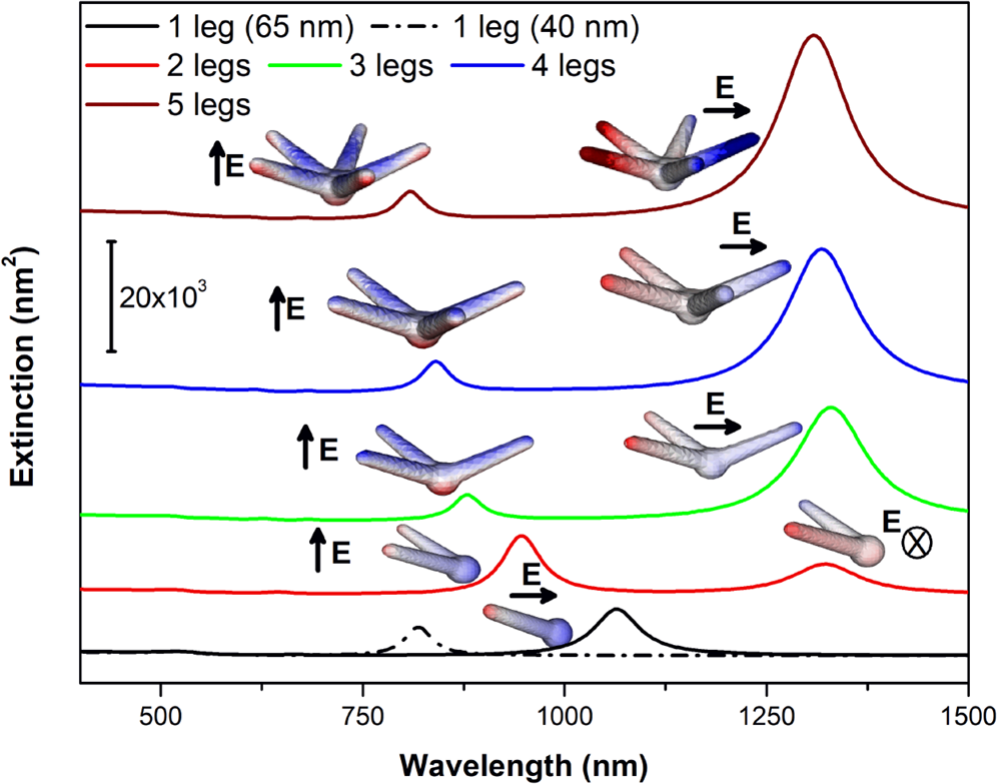
Polarization-averaged extinction spectra for AuNSs with varying numbers of 65 nm upper-plane legs. For the single-leg case, both 40 and 65 nm lengths were considered to reproduce the plasmonic response of a single-leg structure. Charge density distributions and the corresponding incident radiation polarizations are also displayed for the main plasmon modes.

For a single leg, the extinction spectrum exhibits a single dipolar mode arising from coupling between core and leg.^30^ As expected, a shorter leg leads to a higher energy resonance (817 nm for 40 nm vs 1065 nm for 65 nm).

The two-leg structure exhibits two distinct resonances: one blue-shifted (945 nm) and another red-shifted (1321 nm) with respect to the single 65 nm leg case. The lower-energy mode features a nodal *xz*-plane, with the two legs carrying opposite charges, while the higher-energy mode involves charge oscillations extending from the core toward the leg tips. This spectral splitting can be rationalized using a simple system of two coupled harmonic oscillators (see SI file, Figure S2), each representing one leg.^31,32^ The plasmonic coupling between the legs mediated by the core gives rise to two normal modes: a lower-energy symmetric (in-phase) and a higher-energy antisymmetric (out-of-phase) configuration. According to this model, the observed resonances at 945 and 1321 nm correspond, respectively, to the out-of-phase and in-phase coupling of the individual leg plasmonic oscillations. The phase relationship can be interpreted in terms of the *y*-component of the induced dipole moment in each leg.

Similar charge distributions are observed for structures with three, four, and five legs. The high-energy mode retains an out-of-phase, radial-like character, with electron oscillations extending from the core toward the leg tips, while the low-energy mode preserves opposite charge distributions across a nodal plane, corresponding to in-phase coupling. The low-energy resonance remains nearly unchanged in spectral position as the number of legs increases (for example, 1308 nm for five legs, close to 1313 nm for mode #3 in AuNS1). However, its extinction cross-section increases steadily with the number of legs, consistent with an enhanced overall dipole moment.

In contrast, the high-energy mode exhibits a progressive blue shift and a decrease in extinction cross-section with increasing leg number, due to destructive interference between induced dipole components along the *x*- and *y*-axes. Remarkably, the charge density distribution of this mode closely resembles the high-energy resonances observed in the upper half of AuNS1–AuNS4 (particularly modes #1 and #2) indicating strong contributions from these radial-like excitations originating at the nanostar core. Furthermore, its spectral position nearly coincides with that of the 40 nm single-leg resonance, especially for the four- and five-leg configurations.

This spectral overlap supports the interpretation of modes #1 and #2 in AuNS1 and AuNS2 as hybridized plasmon modes, resulting from coupling between the shorter (40 nm) leg and the collective (radial) resonance of the four or five longer legs. Figure 5A illustrates this hybridization scheme,^30^ where the charge distributions of modes #1 and #2 in AuNS1 can be rationalized as linear combinations of the single 40 nm leg mode and the five-leg structure mode, resembling antibonding (out-of-phase coupling) and bonding (in-phase coupling) hybrid states, respectively.

**Figure 5:**
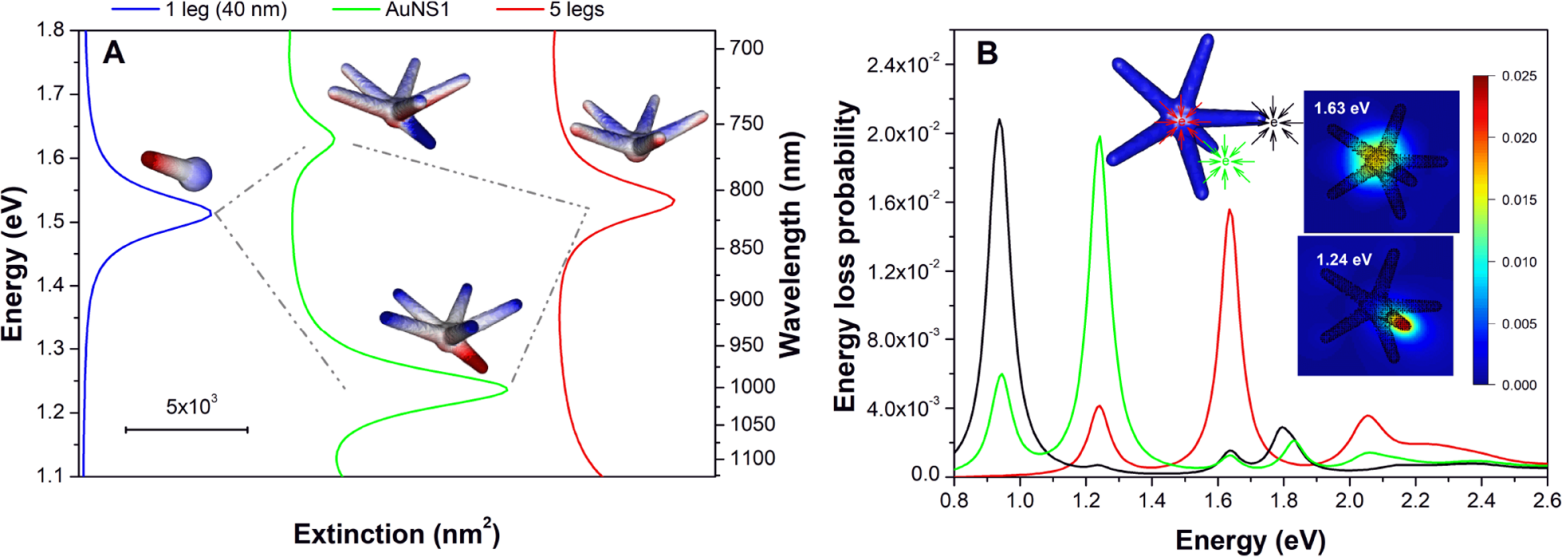
(A) Molecular-orbital-like diagram illustrating the hybridization picture of modes #1 and #2 in AuNS1 (center, green line). These plasmonic modes are described as linear combinations of the resonances of a single 40 nm leg (left, blue line) and a five-leg structure (right, red line). Mode #1 corresponds to the antibonding (out-of-phase) hybrid state, while mode #2 corresponds to the bonding (in-phase) hybrid state. (B) EELS probability spectra for different impact parameters (inset) and the loss probability maps for the modes at 1.24 and 1.63 eV.

Radial plasmonic modes have been investigated in several studies.^33,34^ Schmidt et al. examined radial modes in silver nanodisks of varying diameters using a combined EELS and cathodoluminescence approach.^33^ Due to their vanishing dipole moment, such modes in nanodisks are optically dark and do not efficiently couple to light (except for larger structures, as reported in their work) making EELS a particularly powerful technique for their investigation.^33,35,36^ Following this rationale, we performed EELS simulations to probe the radial-like contribution to plasmonic modes #1 and #2 in AuNS1 (eqs S12 and S13 presented in the SI file). The EELS spectra for three beam positions are shown in Figure 5B: at the nanoparticle core (red) and at the tips of leg #2 and leg #6 (see Figure 1).

When the electron beam is positioned near the tip of leg #2 (a long leg, black curve in Figure 5B), a prominent loss probability is observed at 0.94 eV, corresponding to mode #3. This result is in agreement with experimental investigations.^37^ Under the same excitation conditions, modes #2 (1.24 eV) and #1 (1.63 eV) exhibit considerably lower loss probabilities. Notably, a peak at approximately 1.8 eV appears with significant intensity in the energy-loss spectrum. This mode, together with another at around 2.05 eV, shows very low intensities in the optical extinction spectra of all AuNSs (600–700 nm range). Interestingly, the mode at 2.05 eV is not observed when the beam is positioned near the tip but becomes evident when the probe passes close to the nanoparticle core (red curve).

The most relevant observation under this excitation is the increased loss probability for modes #1 and #2 when the beam passes through the core, particularly for the former. The EELS map for mode #1 at 1.63 eV reveals stronger loss probabilities near the core region, consistent with a radial charge distribution character. Notably, the loss-probability pattern is slightly distorted near the shorter leg, which may arise from out-of-phase coupling, as illustrated schematically in Figure 5A. Conversely, the EELS map for mode #2 shows a much greater contribution when the e-beam is positioned at the tip of the shorter leg alocated at the lower-plane of the icosahedron, highlighting its greater contribution to mode #2.

Overall, these EELS results corroborate our mode assignments and provide a more comprehensive picture of the plasmonic response of AuNSs derived from icosahedral seeds. Figure S5 in the Supporting Information (SI) presents the EELS spectra for a planar AuNS with five legs, for which the radial mode is optically dark but can be readily excited by the electron beam, further supporting our interpretation. The SI also includes the EELS results for AuNS2–AuNS4 (Figure S6).

While AuNS1 was used as the primary example, modes #1 and #2 in AuNS2 can also be interpreted in terms of hybridized states. The differences arise mainly from two factors: (i) the weaker spectral overlap between the single-leg and four-leg structures, and (ii) the orientation of the shorter leg (#5) in AuNS2, which is oriented along the *z*-axis rather than aligned with the general direction of the legs originating from the upper icosahedral plane (Figure 1). In contrast, the shorter leg in AuNS1 is aligned with leg #1, a configuration that may enhance coupling between plasmonic modes. This geometric distinction likely explains the different charge distribution observed for mode #2 in AuNS2 compared with the same mode in AuNS1. EELS simulations for AuNS2 (Figure S6 in the SI file) show that mode #2 exhibits a much larger loss probability than mode #1. These symmetry-related effects require further investigation.

An important question concerns why mode #2 is not observed in AuNS3 and AuNS4. To gain further insight, we performed BEM optical simulations of AuNS1-like structures in which the five upper-plane legs were fixed at 65 nm in length, while the lower-plane leg (leg #6) was varied from 0 to approximately 60 nm. The polarization-averaged extinction spectra for these systems are shown in Figure 6A, focusing on the spectral region encompassing modes #1 and #2.

**Figure 6:**
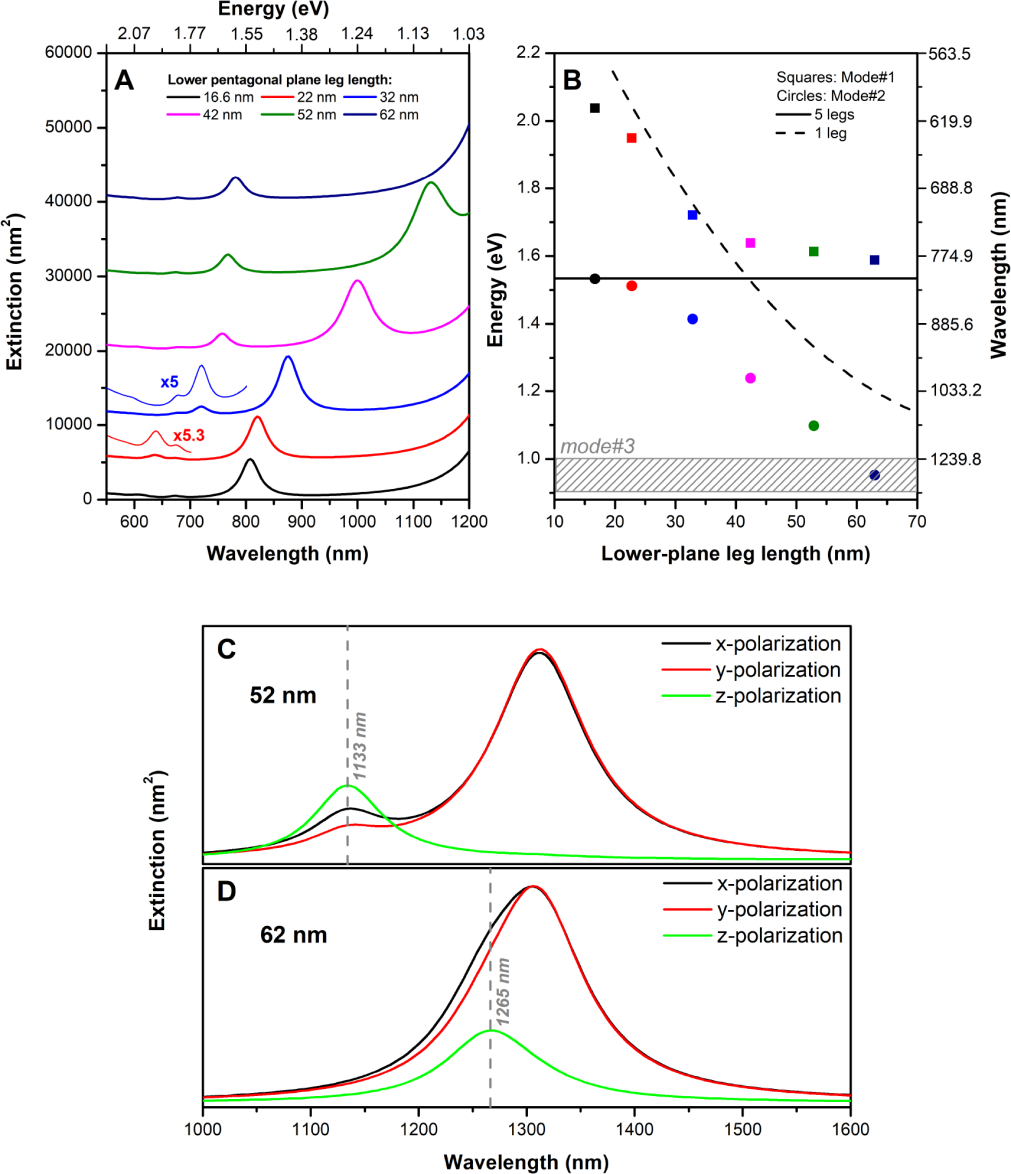
(A) Polarization-averaged extinction spectra for AuNS1-like structures with varying lengths of leg #6. The five legs from the upper plane are modeled with a 65 nm length in all cases. The high-energy regions for leg #6 lengths of 22 and 32 nm are expanded to highlight low-intensity modes. (B) Plasmon energies of mode #1 (squares) and mode #2 (circles) as a function of leg #6 length, compared with the five-leg AuNS structure (absence of leg #6, solid black line) and the single-leg structure (dashed black line). The color scheme reflects the colors of lines in each extinction spectrum. (C, D) show the extinction spectra for different incident polarizations for 52 and 62 nm lengths for leg #6, respectively.

In the absence of leg #6, a prominent peak is observed, associated with the collective charge oscillation from the core to the leg tips, appearing at 808 nm (ca. 1.53 eV, Figure 6A). Increasing the length of leg #6 to 22 nm does not shift this peak significantly, but a new band emerges in the higher energy region (∼636 nm). This resonance lies close to the plasmon mode of a single-leg nanoparticle, as can be observed in Figure 6B, which compares the energies of the observed bands (red and blue circles) with those of a single leg of varying lengths (dashed black line) and of the five-leg AuNS system (solid black line). This result suggests that for short leg #6 lengths, the higher-energy mode has a greater contribution from the single-leg resonance, whereas the lower-energy mode is dominated by the five-leg AuNS radial-like oscillation. It is worth noting that these modes are not strictly independent, as evidenced by their slight redshift relative to the individual modes, indicating plasmonic coupling.

As the length of leg #6 increases, the energy of the single-leg plasmon mode decreases, approaching that of the five-leg AuNS radial mode. For leg #6 lengths of 40 nm or larger, an inversion of mode ordering occurs: the plasmon mode of the single-leg structure becomes less energetic than that of the five-leg system. Under these conditions, stronger coupling appears to set in, as evidenced by the spectral shifts observed in the AuNS1 modes relative to those of the five-leg and single-leg configurations. Further evidence of strong coupling is provided by the energy splitting observed in the resonance curves, indicating an avoided-crossing behavior characteristic of the strong-coupling regime.^38,39^ Figure S3 in the SI compares the resonance energies of modes #1 and #2 with the expected evolution from weak to strong coupling, clearly highlighting the avoided-crossing behavior in AuNS1.

Interestingly, for increasing leg #6 lengths, modes #2 and #3 bands overlap, as indicated by the shaded region in Figure 6B (band position estimated from full width at half-maximum, FWHM). For a leg #6 length of 52 nm, both mode #2 and mode #3 are clearly visible in the extinction spectrum (Figure 6C). Importantly, mode #2 appears mainly under *z*-polarized excitation, consistent with its dipole moment being oriented along the *z*-axis. At longer leg #6 lengths (Figure 6D), modes #2 and #3 overlap, explaining why mode #2 is not readily observed in AuNS3 and AuNS4, where all legs have the same 65 nm length. In these structures, mode #2 is accessible for incident radiation polarized along *z*, which also accounts for the appearance of a band in the mode #3 region under *z*-polarization for AuNS2 and AuNS3.

### Size Dependence

The discussions above suggest that the clear observation of mode #2 in the extinction spectrum depends on the presence of at least one shorter lower-plane leg (or a leg grown in the −*z* direction, as in AuNS2). From a synthetic perspective, achieving such a high degree of control over both the number and the relative lengths of legs is extremely challenging, and the samples most likely contain significant variations in these parameters across different AuNSs. Consequently, the contribution of mode #2 to the experimental extinction spectrum is expected to be reduced. Figure 7 compares an experimental extinction spectrum with BEM simulations, where AuNS1 was chosen as representative of the overall optical response of the nanostars. Details for the gold nanostar synthesis are presented in the SI file.

**Figure 7:**
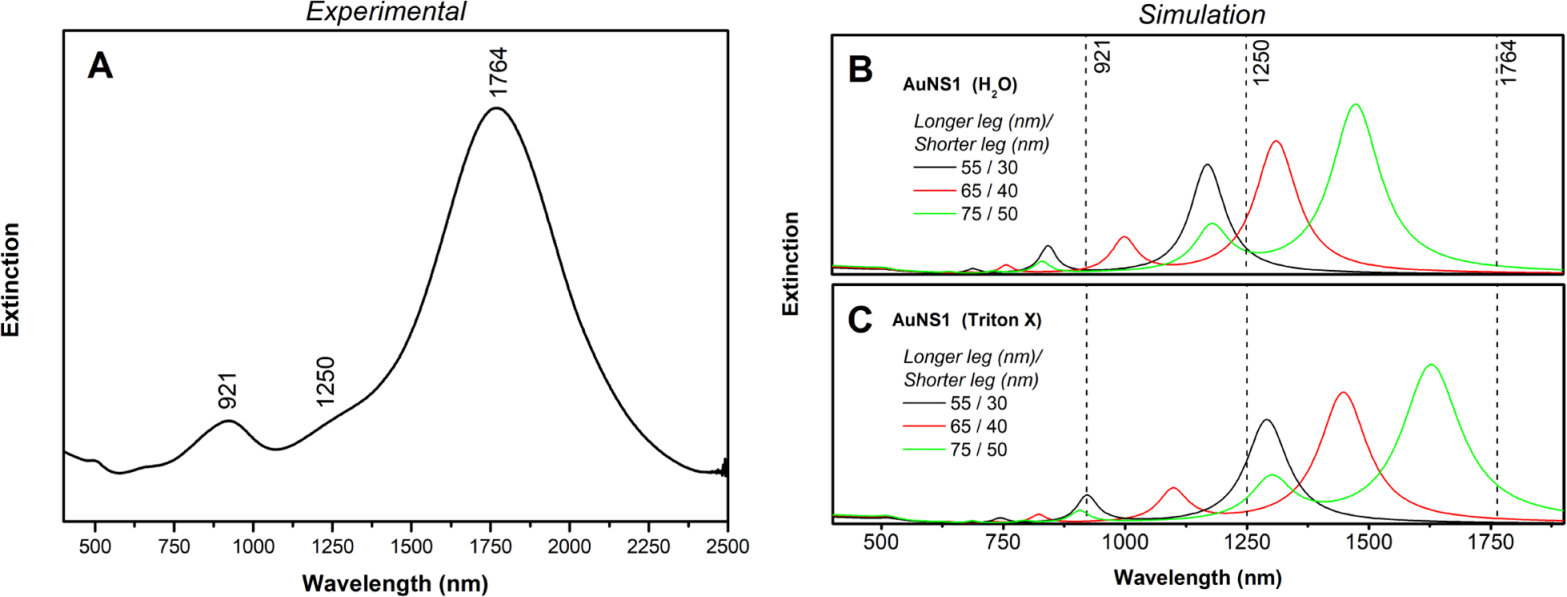
Comparison between experimental (A) and BEM-simulated extinction spectra. The BEM simulations were performed for AuNSs with the same structure as AuNS1, varying all leg lengths by ±10 nm. Two dielectric environments were considered: water (B) and Triton X-100 (C), one of the residual species from the synthesis.

The experimental extinction spectrum exhibits two prominent bands around 921 and 1764 nm, together with a shoulder near 1250 nm. This spectral profile is consistent with the presence of three modes (modes #1, #2, and #3) identified in the BEM simulations. The most intense peak at 1764 nm displays a broad line shape, consistent with a distribution of leg sizes. The shoulder around 1250 nm may be associated with mode #2, arising from AuNSs containing at least one shorter lower-plane leg. However, we cannot exclude the possibility that this shoulder originates from nanostars with overall shorter legs, for which mode #3 would shift to lower wavelengths.

This effect can be schematically illustrated by simulations of AuNS1 structures with leg lengths varied by ±10 nm, considering water as the surrounding dielectric medium. Although the simulated resonances in water occur near 921 and 1250 nm, the calculated bands are blueshifted relative to the experimental data. A likely reason for this discrepancy is the use of water as the dielectric environment in the BEM simulations. Triton X-100 (refractive index 1.42), a residue from the synthesis, should also be considered for more quantitative descriptions of the plasmonic resonances. Figure 7 also includes the results obtained when replacing water with Triton X-100 as the surrounding medium. The agreement with the experimental spectrum improves, although the lower-energy bands remain blueshifted. This residual shift may result from (i) an ensemble of nanostars with longer legs, and/or (ii) inefficiencies in the dielectric function for the description of such high wavelength region.

The first hypothesis would affect all modes equally, which is not observed. Therefore, it is more plausible that the remaining discrepancies arise from limitations in the dielectric data. The optical constants from Palik^25^ and Johnson and Christy^23^ exhibit higher imaginary components above 1000 nm compared to more recent compilations, such as that of Babar and Weaver.^40^ Adjusting the dielectric function may thus improve quantitative agreement with experimental data—particularly for mode #3—without altering the overall physical interpretation.

### Near-Field Enhancements

A key feature of metallic nanostructures is the strong localization of electromagnetic fields arising from plasmonic excitations, which plays a central role in enhanced spectroscopies such SERS. The SERS enhancement factor *F*—defined as the ratio between the SERS and Raman intensities for a given molecule—can be estimated within the *E* ^4^-approximation as^41–43^

$$F\left(\omega ,r\right)={\left(\frac{E\left(\omega ,r\right)}{E_{0}\left(\omega \right)}\right)}^{4}$$ 3

where *E*(ω, *r*) is the local electric field at position *r* and *E*_0_(ω) is the incident field amplitude at frequency ω. Figure 8 shows the SERS enhancement factor distributions at 1 nm distance from the AuNS1 surface. As expected, the field enhancements are predominantly localized at the leg tips. Mode #1 exhibits the lowest enhancement values, consistent with an out-of-phase interaction between plasmonic modes (dark mode). In contrast, modes #2 and #3 display the strongest local SERS enhancement factors, reaching values on the order of 10^8^, comparable to those typically observed in hot spots formed by closely spaced nanoparticles. The field distribution for mode #2 reveals a greater contribution from the shorter 40 nm leg (#6), consistent with EELS probability maps, that shows a greater contribution of this leg to the plasmonic mode (Figure 5B).

**Figure 8:**
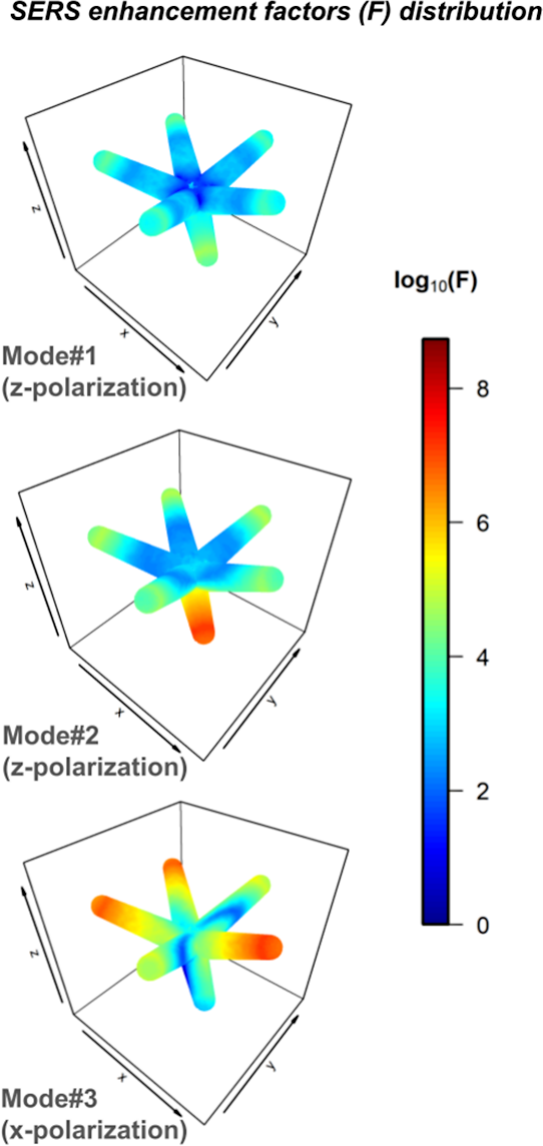
SERS enhancement factor distributions calculated at 1 nm distance from AuNS1 surface for modes #1–#3. The results are presented log-scale. For modes #1 and #2, *z*-polarization was chosen, whereas *x*-polarization was used for F calculation at mode #3.

Although the extinction cross-section of mode #3 is considerably larger than that of mode #2, their maximum field enhancements are comparable. The difference arises from the number of legs contributing to the enhancement: in mode #3, multiple legs are involved, leading to a greater number of hot spot regions. This suggests that mode #3 could provide higher overall SERS sensitivity, since more molecules adsorbed on the surface of different legs may experience strong field enhancement simultaneously.

Similar near-field distributions are observed for AuNS2, AuNS3, and AuNS4 (Figure S7), although lower maximum near-field enhancements are obtained, especially for mode #1. This behavior can be attributed to the orientation of leg #5 (Figure 1), which leads to stronger interference effects, thereby reducing the overall field enhancement. Figure S8 also presents the relative maximum enhancements for AuNS1 as a function of leg length, following the same variations shown in Figure 7, indicating a higher enhancement for a leg length of 65 nm. Further investigations are still required to fully describe the effect of leg length on the SERS properties of such nanostars.

## Conclusions

The optical properties of AuNSs simulated by the BEM method reveal strong coupling between plasmonic modes supported by individual legs, giving rise to both in-phase and out-of-phase interactions. The latter are manifested at higher energies in the extinction spectra, with mode #1 (Figure 2) being particularly sensitive to structural variations. In particular, increasing the number of legs results in a pronounced blue-shift of this mode, indicating its potential use as a probe of leg multiplicity in specific synthesis protocols. The dark nature of such mode, as corroborated by EELS simulations, may be explored for different fundamental investigations of surface plasmons, such as Fano resonances and plasmonic photocatalysis.^35,44,45^

At lower energies, in-phase coupling of all legs dominates the plasmonic response. This mode produces intense local field enhancements, highly relevant for surface-enhanced spectroscopies such as SERS. The calculated SERS enhancement factors are comparable to those of hot spots formed in nanoparticle dimers, with the added advantage that the amplified fields are distributed across multiple leg tips, thereby increasing the sensitivity of SERS detection for molecules adsorbed on the nanoparticle surface.

Structurally, AuNSs can be rationalized as resulting from leg growth at adjacent vertices through a surfactant-driven unzipping mechanism, which favors leg formation from vertices on the same plane of the icosahedral core. During the synthesis, however, additional legs may emerge from the lower plane. In this work, we investigated systems with up to two lower-plane legs, which give rise to a distinct intermediate-energy mode (mode #2). This mode is highly sensitive to the length of the additional leg and exhibits local field enhancements comparable to those of mode #3, although with a more localized distribution concentrated on the extra leg.

These findings provide fundamental insights into the plasmonic response of Au nanostars produced from icosahedral Au seeds and open up new perspectives for tailoring their optical properties for different applications such as ultrasensitive plasmon-enhanced spectroscopies.

## Supplementary Material



## Notes

Detailed discussions of the BEM formalism and experimental methods, extinction spectra simulations for AuNS1 at different polarizations and radiation propagation directions, coupled-oscillator analyses of plasmonic mode interactions in AuNSs, and additional results from EELS simulations and SERS enhancement calculations (PDF)
