# Supplementary material for: Plasmonic Properties of Icosahedral-Seeded Gold Nanostars
Source: J Phys Chem C Nanomater Interfaces. 2026 Jan 20;130(4):1609–18. doi: 10.1021/acs.jpcc.5c07756 (PMC12862814; doi:10.1021/acs.jpcc.5c07756)
Supplement: Supplementary file 1 [file jp5c07756_si_001.pdf]

Supporting information to: “PLASMONIC PROPERTIES OF ICOSAHEDRAL-SEEDED GOLD NANOSTARS”

Oliver Leal de Castro Prioli,† Debora Ferrari,‡ Laura Fabris,‡ Daniel Ugarte,¶ and Diego Pereira dos Santos*,†

†*Institute of Chemistry, State University of Campinas, Campinas 13083-862, Brazil*

‡*Department of Applied Science and Technologies, Politecnico di Torino, Turin 10129, Italy*

¶*Institute of Physics Gleb Wataghin, State University of Campinas, Campinas 13083-859, Brazil*

E-mail: santosdp@unicamp.br

Contents

- Boundary element method (BEM) simulations theory
- Experimental details
- Extinction spectra simulations
- Coupled harmonic oscillators
- EELS simulations
- Near-field simulations: SERS enhancement factors

Boundary element method (BEM) theory

The optical response of gold nanostars (AuNSs) was investigated using BEM,[1] as implemented in the MNPBEM17 toolbox developed by Hohenester and Trügler.[2] In BEM, the interaction of the nanoparticle with the incident electromagnetic field is described in terms of its surface elements (∂V), reducing Maxwell's equations to boundary integrals. In this approach, the electric ($\mathbf{E}$) and magnetic ($\mathbf{H}$) fields are expressed through the scalar (Φ) and vector ($\mathbf{A}$) potentials:

$$\mathbf{E} = ik\mathbf{A} - \nabla\Phi \tag{S1}$$

$$\mathbf{H} = \frac{1}{\mu}\nabla \times \mathbf{A} \tag{S2}$$

where $k = \sqrt{\varepsilon}\,\omega/c$ is the wavevector in a medium of dielectric function $\epsilon(\omega)$ and magnetic permeability μ. The scalar and vector potentials at position $\mathbf{r}$ are obtained from the boundary integrals:

$$\Phi(\mathbf{r}) = \int_{\partial V} G(|\mathbf{r} - \mathbf{s}'|)\sigma(\mathbf{s}')\, da' + \Phi_{\text{ext}}(\mathbf{r}) \tag{S3}$$

$$\mathbf{A}(\mathbf{r}) = \int_{\partial V} G(|\mathbf{r} - \mathbf{s}'|)\mathbf{h}(\mathbf{s}')\, da' + \mathbf{A}_{\text{ext}}(\mathbf{r}) \tag{S4}$$

with G describing the Green's function

$$G(r) = \frac{e^{ikr}}{r} \tag{S5}$$

σ and $\mathbf{h}$ are the surface charge and current densities, and Φ_{ext} and $\mathbf{A}_{\text{ext}}$ the external potentials, representing the incident radiation. The central point of BEM is the calculation of such surface charges and currents, from which all optical properties can be derived from

Eqs. S1, S2, S3 and S4.

The scalar potential is continuous across the boundary δV, i.e. $\Phi_1 = \Phi_2$, where the numbers 1 and 2 are used to denote the internal (j = 1) and external (j = 2) media in respect to the boundary. This continuity can be described as:

$$\int_{\partial V_1} G_1(\mathbf{r}-\mathbf{s}')\sigma_1(\mathbf{s}')\,da' + \Phi_{\text{ext},1}(\mathbf{r}) = \int_{\partial V_2} G_2(\mathbf{r}-\mathbf{s}')\sigma_2(\mathbf{s}')\,da' + \Phi_{\text{ext},2}(\mathbf{r}) \tag{S6}$$

Eq. S6 can be rewritten in the form $\mathbf{G_1}\boldsymbol{\sigma_1} - \mathbf{G_2}\boldsymbol{\sigma_2} = \Phi_{\text{ext},2} - \Phi_{\text{ext},1}$, which is particularly useful for numerical implementations, where the integration is substituted by a summation over the discrete surface elements:

$$\int_{\partial V_j} G_j(\mathbf{r}-\mathbf{s}')\sigma(\mathbf{s}')\,da' = \sum_i G_{j,i}\sigma_{j,i}\Delta A_{j,i} = \mathbf{G_j}\boldsymbol{\sigma_j} \tag{S7}$$

where the right-hand side of Eq. S7 is a matrix description of the problem in medium j after the discretization of the nanoparticle surface.

The inclusion of continuity for the components of the electric and magnetic fields tangential to the surface leads to a system of linear equations from which the surface charge and current density matrices can be calculated. For a more in-depth view of the mathematical framework the reader is referred to the work done by García de Abajo et. al.[1,3]

The scattering and extinction powers (P_{sca} and P_{ext}, respectively) are calculated using the following equations:

$$P_{\text{sca}} = n_b \oint_{\mathcal{S}} \Re\left[\mathbf{n}\cdot(\mathbf{E}\times\mathbf{B}^*)\right]\,da, \tag{S8}$$

$$P_{\text{ext}} = -\frac{1}{n_b}\oint_{\mathcal{S}} \Re\left[\mathbf{n}\cdot(\mathbf{E}\times\mathbf{B}^*_{\text{inc}} + \mathbf{E}^*_{\text{inc}}\times\mathbf{B})\right]\,da, \tag{S9}$$

where n_b is the refractive index of the embedding medium, $\mathbf{E}$ and $\mathbf{B}$ are the scattered

electromagnetic fields, $\mathbf{E}_{\rm inc}$ and $\mathbf{B}_{\rm inc}$ are the incident fields, and the integration extends over a closed surface $\mathcal{S}$ enclosing the particle.

Finally, the cross-sections can be calculated using the incident radiation intensity (I_{inc})

$$C_{\rm sca} = \frac{P_{\rm sca}}{I_{\rm inc}}, \qquad C_{\rm ext} = \frac{P_{\rm ext}}{I_{\rm inc}}, \qquad C_{\rm abs} = C_{\rm ext} - C_{\rm sca}. \tag{S10}$$

where C_{abs} is the absorption cross-section.

Electron energy loss spectroscopy simulations are performed within the same framework of Eqs. S3 and S4, but with the external potentials described by a high velocity (v) electron beam:[4]

$$\phi_{\rm ext}(\mathbf{r}) = -\frac{2}{v\varepsilon_j} K_0\left(\frac{q|\mathbf{R}-\mathbf{R}_0|}{\gamma_j}\right) e^{iq(z-z_0)}, \qquad \mathbf{A}_{\rm ext}(\mathbf{r}) = \varepsilon_j \frac{\mathbf{v}}{c}\phi_{\rm ext}(\mathbf{r}) \tag{S11}$$

where $\mathbf{R}$ describes a position in the xy-plane (assuming a beam propagating in the z-direction). $\mathbf{R}_0$ is the beam impact parameter (beam position). K_0 is the modified Bessel function of order zero and the parameter γ_j is related to the dielectric function of jth material through $\gamma_j = (1-\epsilon_j v^2)^{-\frac{1}{2}}$. It is important to note that this formalism assumes that the electron energy loss (ΔE) process through interaction with the induced electric fields ($\mathbf{E}_{\rm ind}$) in the simulated system is a small perturbation. In this case it can be computed by:

$$\Delta E = \int_0^\infty \hbar\omega\, \Gamma_{\rm EELS}(\mathbf{R},\omega)\, d\omega. \tag{S12}$$

where $\Gamma_{\rm EELS}$ is the loss probability:

$$\Gamma_{\rm EELS}(\mathbf{R},\omega) = \frac{e}{\pi\hbar\omega}\int \Re\left\{e^{-i\omega t}\,\mathbf{v}\cdot\mathbf{E}_{\rm ind}(\mathbf{r}(t),\omega)\right\} dt + \Gamma_{\rm bulk}(\omega) \tag{S13}$$

$\Gamma_{\rm bulk}$ describes the bulk loss probability and the first term on the right hand side is the surface loss probability, which is presented throughout the manuscript.

Experimental details

The seed solution was obtained by adding 0.6 mL of a freshly prepared, ice-cold 0.01 M $NaBH_4$ solution to 10 mL of an aqueous solution containing 0.15 M Triton X-100 and 0.25 mM $HAuCl_4$. Upon $NaBH_4$ addition, the solution immediately changed color from pale yellow to orange. The mixture was stirred for 7 minutes and stored at 4 °C for no longer than 3 hours prior to use.

The growth solution consisted of an aqueous solution of 0.15 M Triton X-100 and 0.5 mM $HAuCl_4$. The following reagents were rapidly added to this solution: 0.04 mL of 0.789 M L-ascorbic acid, 0.5 mL of 3.94 mM $AgNO_3$, and 0.014 mL of the seed solution. The resulting mixture was stirred for 7 minutes, then centrifuged at 4000 $\times$ g for 10 minutes, and finally redispersed in Milli-Q water (18.2 MΩ.cm).

The extinction spectra of six-branched gold nanostars (6bGNS) were acquired using a Cary 5000 UV-Vis-NIR Spectrophotometer (Agilent Technologies) with an extended-range high-performance quartz glass cuvette (optical path length: 1 mm). The source changeover wavelength was set to 299 nm, with an averaging time of 0.1 s. Spectra were collected over a wavelength range of 300-2500 nm.

Extinction spectra simulations

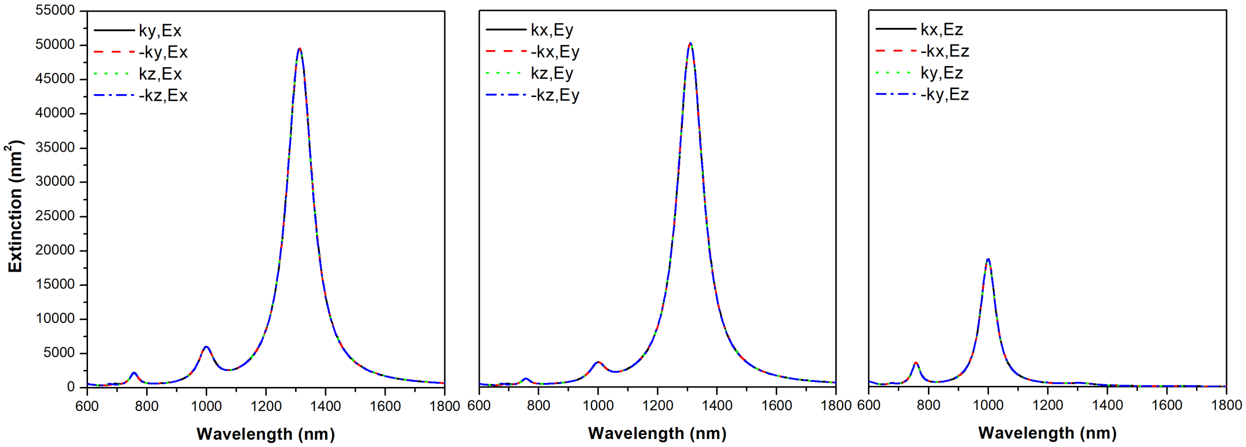

Figure S1: Extinction spectra for different incident field polarizations: along x (left panel), y (midle panel) and z (right panel). For each polarization, different radiation propagation directions are considered, represented by the wavevector, **k**, components.

Coupled harmonic oscillators

A system of two coupled harmonic oscillators (Fig. S2) can be described by the following set of equations:[5]

$$\begin{cases} \ddot{x}_1(t) + \gamma_1 \dot{x}_1(t) + \omega_1^2 x_1(t) - \chi_{12}^2 x_2(t) = Ae^{-i\omega t} \\ \ddot{x}_2(t) + \gamma_2 \dot{x}_2(t) + \omega_2^2 x_2(t) - \chi_{12}^2 x_1(t) = 0 \end{cases} \tag{S14}$$

where x_i describes the ith particle's motion that oscillates with a characteristic frequency ω_i dictated by the constant force k_i. This motion is assumed to be dumped by constant of γ_i. The two oscillators are connected by a force constant k_{12} that induces a coupling constant given by χ_{12}. A and ω are the amplitude and frequency of the external perturbation.

Assuming a harmonic motion, the solutions for x_i can be described by:

$$x_i = C_i(\omega) e^{-iwt} \tag{S15}$$

where the amplitudes C_i can be used to model the shape of the extinction spectrum. Fig. S2 presents the comparison between the extinction spectrum for a two leg AuNS with the coupled oscillator model for the following parameters:

Table S1: Parameters used in the coupled oscillator model of Fig. S2.

**Energy (eV)**	**Dumping constant (eV)**	**Coupling constant (eV)**
$\omega_1 = 1.16$	$\gamma_1 = 0.1$	$\chi_{12} = 0.45$
$\omega_2 = 1.16$	$\gamma_2 = 0.1$	

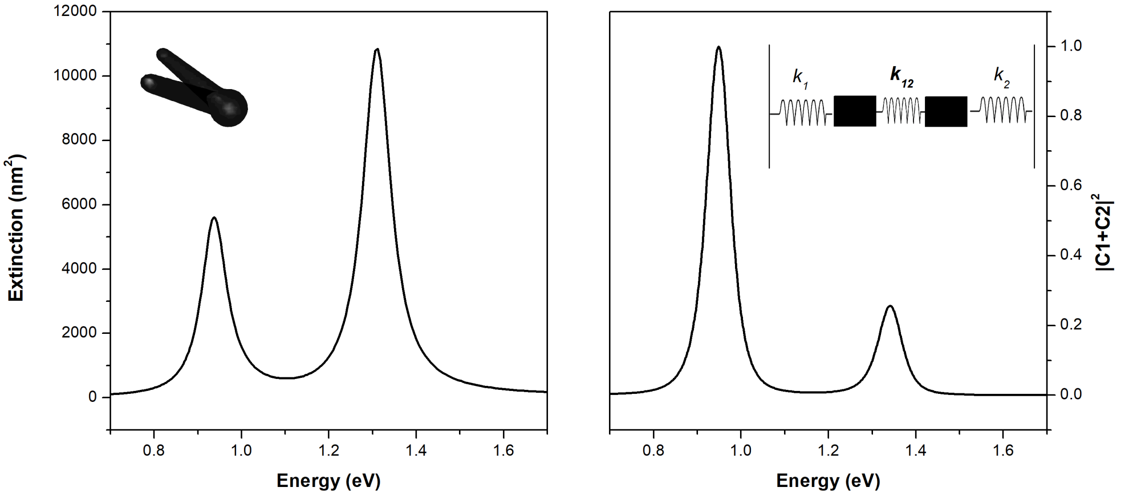

Figure S2: Comparison between the simulated extinction spectrum for an AuNS with two legs (left) and the amplitude spectrum of a system of two coupled harmonic oscillators (right).

Table S2: Parameters used in the coupled oscillator model of Fig. S3. The oscillators in considered in Fig. S3 are the upper-plane 5 leg-system and the lower-plane single-leg system of AuNS1 (see Fig. 5 in the main paper). The parameter L is the lower-plane single-leg length in nm (Fig. 6 in the main paper).

**Energy (eV)**	**Dumping constant (eV)**	**Coupling constant (eV)**
$\omega_1 = 1.53$	$\gamma_1 = 0.1$	$\chi_{12} = 0.1 - 1.0$
$\omega_2 = 2.89 - 0.04L + (2.59 \times 10^{-4})L^2$	$\gamma_2 = 0.2$	

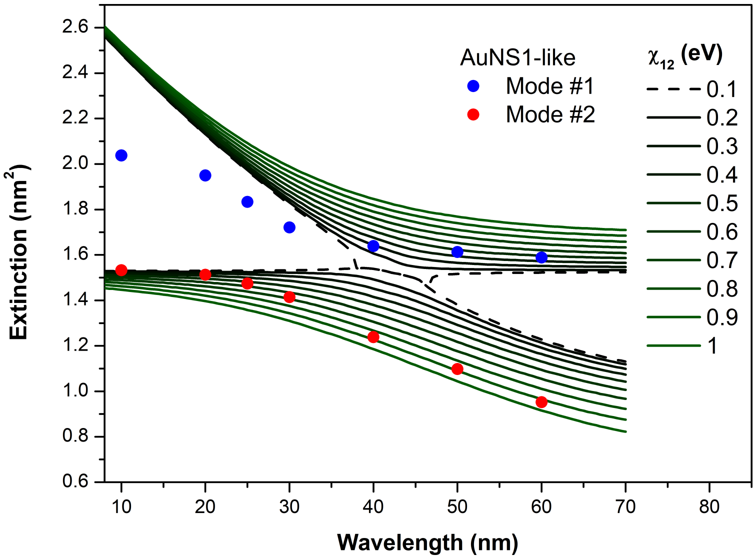

Figure S3: Energy splitting of coupled oscillators as function of coupling strength (χ_{12}). The two oscillators here are taken as the single-leg (of varying lengths) and the collective upper-plane 5 legs mode (fixed 65 nm length for each leg). In the same figure are presented the energies of modes #1 (blue circles) and #2 (red circles) as function of lower-plane leg length in an AuNS1-like structure. At lower coupling constant, it can be observed a crossing between high and low energy modes. As the coupling increases it can be observed an avoided crossing behavior, also noticed in AuNS1 simulated data.

EELS simulations

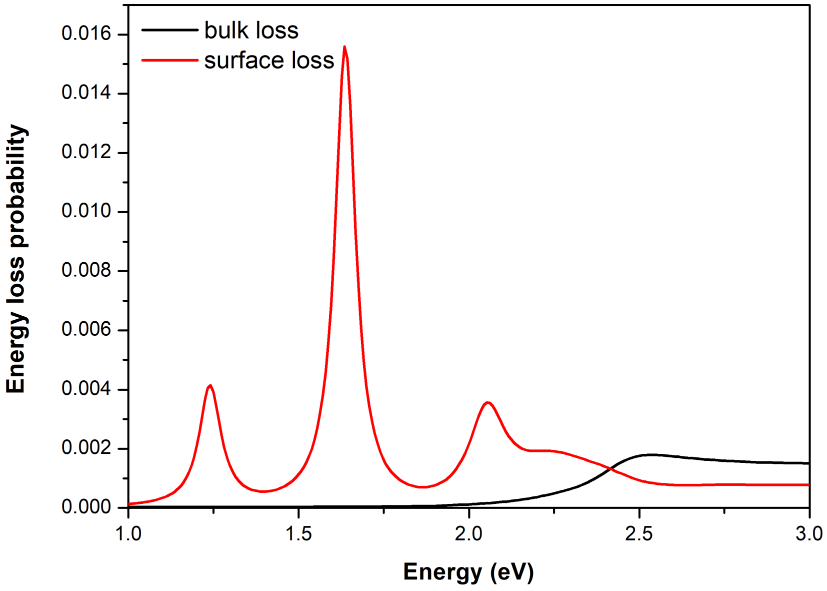

Figure S4: Comparison between surface loss and bulk loss for AuNS1 with impact parameter at the core center.

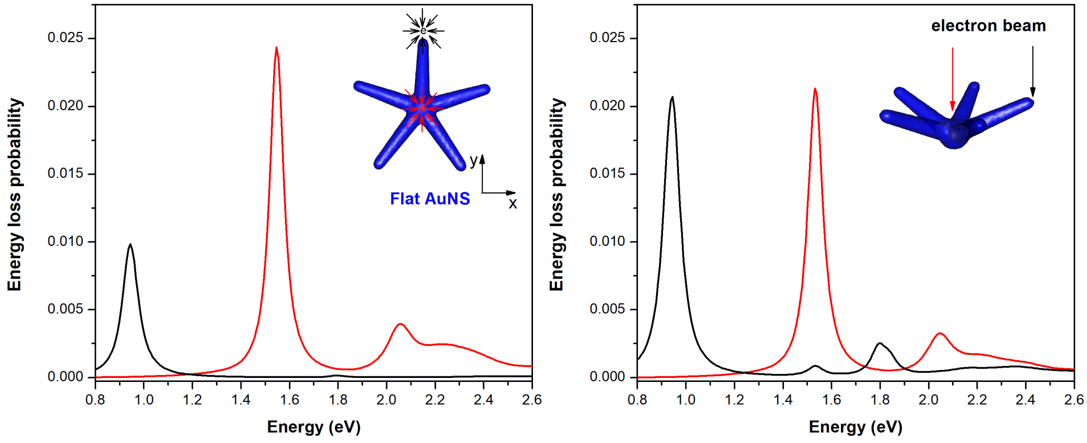

Figure S5: Simulated electron energy loss spectra (EELS) for five leg AuNSs: planar (left) and one driven by the icosahedral seed vertices orientation (right). The loss spectra were simulated at different impact parameters, as indicated by the insets, which illustrates the electron beam positions relative to the nanoparticles.

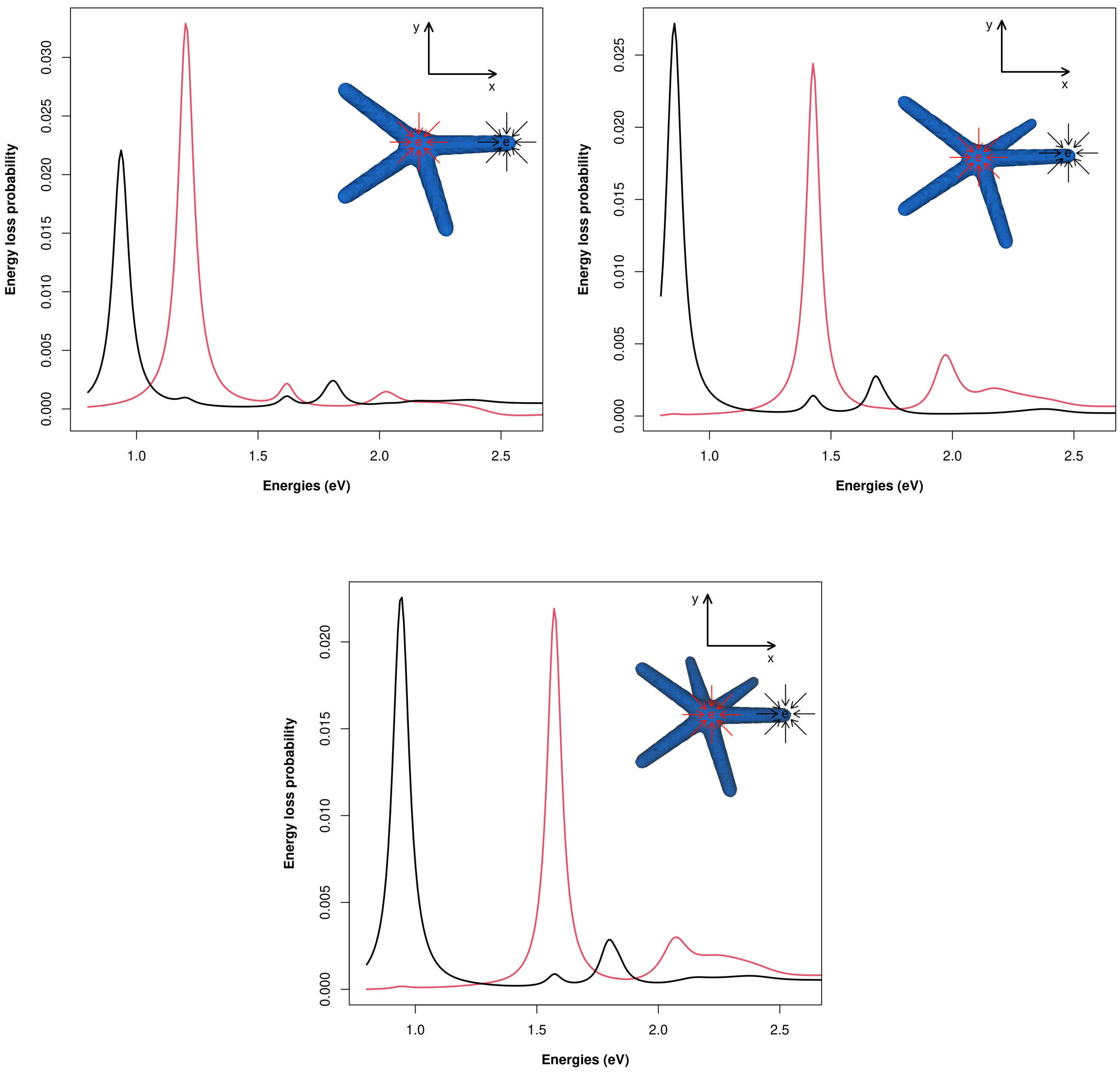

Figure S6: Simulated energy loss spectra (EELS) for five- and six-legged AuNSs (top left: AuNS2, top right: AuNS3 and bottom: AuNS4). The spectra were simulated using different impact parameters: in the core of the AuNS, and in the tip of one of its legs.

Near-field simulations: SERS enhancement factors

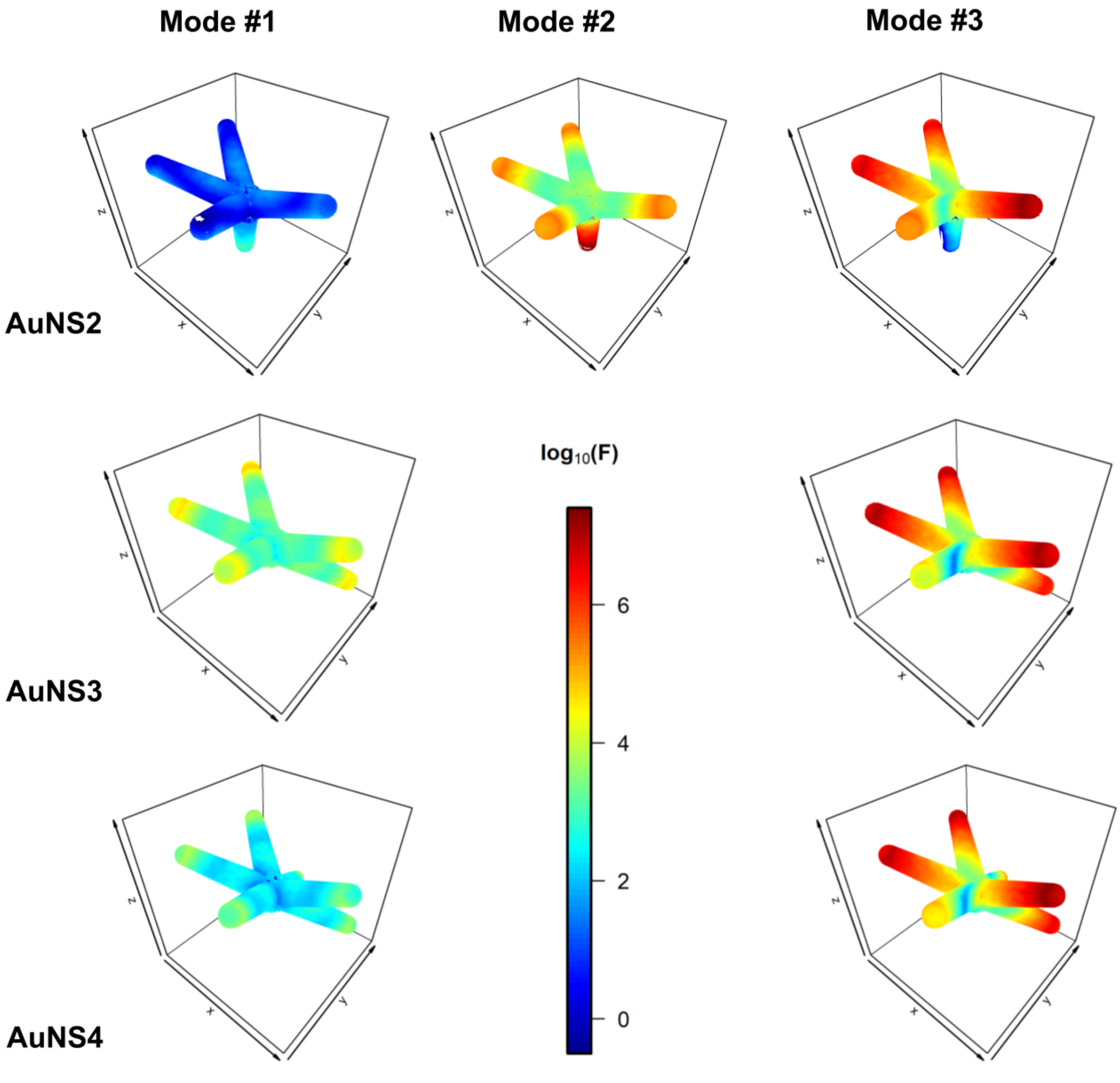

Figure S7: SERS enhancement factor distributions calculated at 1 nm distance from AuNS2, AuNS3 and AuNS4 surface for modes #1, #2 (only AuNS2) and #3. The results are presented log-scale. For modes #1 and #2, z-polarization was chosen, whereas x-polarization was used for F calculation at mode#3.

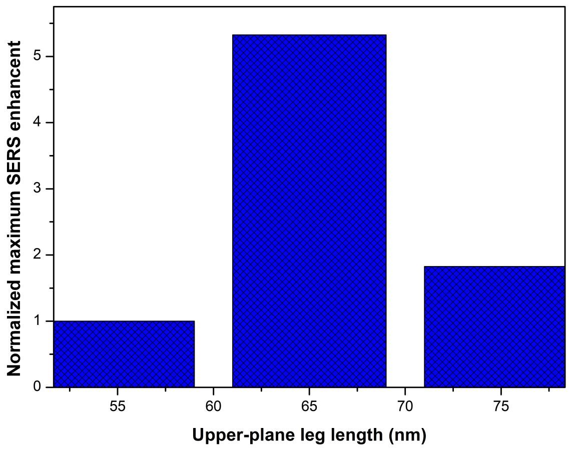

Figure S8: Maximum enhancement factors for x-polarized incident field for AuNS1-like structures with upper-leg sizes of 55, 65 and 75 nm. The data is presented in normalized form with respect to the smallest value for direct comparison.

References

(1) de Abajo, F. J. G.; Howie, A. Retarded field calculation of electron energy loss in inhomogeneous dielectrics. *Phys. Rev. B* **2002**, *65*, 115418.

(2) Hohenester, U.; Trügler, A. MNPBEM – A Matlab toolbox for the simulation of plasmonic nanoparticles. *Computer Physics Communications* **2012**, *183*, 370–381.

(3) Myroshnychenko, V.; Rodríguez-Fernández, J.; Pastoriza-Santos, I.; Funston, A. M.; Novo, C.; Mulvaney, P.; Liz-Marzán, L. M.; García de Abajo, F. J. Modelling the Optical Response of Highly Faceted Metal Nanoparticles with a Fully 3D Boundary Element Method. *Advanced Materials* **2008**, *20*, 4288–4293.

(4) Hohenester, U. Simulating electron energy loss spectroscopy with the MNPBEM toolbox. *Computer Physics Communications* **2014**, *185*, 1177–1187.

(5) Souza, K. S.; Teixeira-Neto, E.; Temperini, M. L. A.; dos Santos, D. P. Interplay between Near-Field Properties and Au Nanorod cluster Structure: Extending Hot Spots for

Surface-Enhanced Raman Scattering. *Journal of the Brazilian Chemical Society* **2019**, *30*, 2624–2633.
